# A simpler method of preprocessing MALDI-TOF MS data for differential biomarker analysis: stem cell and melanoma cancer studies

**DOI:** 10.1186/1559-0275-8-14

**Published:** 2011-09-19

**Authors:** Dong L Tong, David J Boocock, Clare Coveney, Jaimy Saif, Susana G Gomez, Sergio Querol, Robert Rees, Graham R Ball

**Affiliations:** 1 The John van Geest Cancer Research Centre, School of Science and Technology, Nottingham Trent University, Clifton Lane, Nottingham, NG11 8NS, UK; 2 Anthony Nolan Cell Therapy Centre, Nottingham Trent University, Nottingham, NG11 8NS, UK

**Keywords:** MALDI-TOF, MS profiling, raw data, data preprocessing, stem cell, melanoma

## Abstract

**Introduction:**

Raw spectral data from matrix-assisted laser desorption/ionisation time-of-flight (MALDI-TOF) with MS profiling techniques usually contains complex information not readily providing biological insight into disease. The association of identified features within raw data to a known peptide is extremely difficult. Data preprocessing to remove uncertainty characteristics in the data is normally required before performing any further analysis. This study proposes an alternative yet simple solution to preprocess raw MALDI-TOF-MS data for identification of candidate marker ions. Two in-house MALDI-TOF-MS data sets from two different sample sources (melanoma serum and cord blood plasma) are used in our study.

**Method:**

Raw MS spectral profiles were preprocessed using the proposed approach to identify peak regions in the spectra. The preprocessed data was then analysed using bespoke machine learning algorithms for data reduction and ion selection. Using the selected ions, an ANN-based predictive model was constructed to examine the predictive power of these ions for classification.

**Results:**

Our model identified 10 candidate marker ions for both data sets. These ion panels achieved over 90% classification accuracy on blind validation data. Receiver operating characteristics analysis was performed and the area under the curve for melanoma and cord blood classifiers was 0.991 and 0.986, respectively.

**Conclusion:**

The results suggest that our data preprocessing technique removes unwanted characteristics of the raw data, while preserving the predictive components of the data. Ion identification analysis can be carried out using MALDI-TOF-MS data with the proposed data preprocessing technique coupled with bespoke algorithms for data reduction and ion selection.

## 1. Introduction

Matrix-assisted laser desorption/ionisation mass spectrometry (MALDI MS) based proteomics is a powerful screening technique for biomarker discovery. Recent growth in personalised medicine has promoted the development of protein profiling for understanding the roles of individual proteins in the context of amino status, cellular pathways and, subsequently response to therapy. Frequently used ionisation methods in recent MS technologies include electrospray ionisation (ESI), surface-enhanced laser desorption/ionisation (SELDI) and MALDI. Reviews on these methods can be found in the literature [1,2]. One of the commonly used mass analyser techniques in proteomic MS analysis is time-of-flight (TOF), the analysis based on the time measurement for an ion (i.e. signal wave) to travel along a flight tube to the detector. This time representation can be translated into mass to charge ratio (*m/z*) and therefore the mass of the analyte. Data can be exported as a list of values (*m/z *points) and their relative abundance (intensity or mass count).

Typical raw MS data contains a range of noise sources, as well as true signal elements. These noise sources include mechanical noise that caused by the instrument settings, electronic noise from the fluctuation in an electronic signal and travel distance of the signal, chemical noise that is influenced by sample preparation and sample contamination, temperature in the flight tube and software signal read errors. Consequently, the raw MS data has potential problems associated with inter- and intra-sample variability. This makes identification/discovery of marker ions relevant to a sample state difficult. Therefore, data preprocessing is often required to reduce the noise and systematic biases in the raw data before any analysis takes place.

Over the years, numerous data preprocessing techniques have been proposed. These include baseline correction, smoothing/denoising, data binning, peak alignment, peak detection and sample normalisation. Reviews on these techniques can be found in the literature [3-7].

A common drawback of these preprocessing techniques is that they normally involve several steps [8,9] and require different mathematical approaches [10] to remove noise from the raw data. Secondly, most of the publicly available preprocessing techniques focuses on either SELDI-TOF MS, often on intact proteins at low resolution compared to modern instrumentation [3,11] or liquid chromatography (LC) MS [12-14]. These existing preprocessing techniques have limited functions which can be applied to high resolution MALDI-TOF MS peptide data.

This paper proposes a simple preprocessing technique aiming at solving the inter- and intra-sample variability in raw MALDI-TOF MS data for candidate marker ion identification. In the proposed preprocessing technique, the data were aligned and binned according to the global mean spectrum. The region of a peak was identified based on the magnitude of the mean spectrum. One of the main advantages of this technique is that it eliminated the fundamental argument on the uncertainty of the lower and upper bounds of a peak. The preprocessed data is then analysed using bespoke machine learning methods that are capable for handling noisy data. The panel of candidate marker ions is produced based on their predictive power of classification.

For the remainder of this paper, we will first discuss the signal processing related problems associated with MALDI-TOF MS data based on the instrumentation supplied by Bruker Daltonics. We then describe the data sets and the methodology for signal processing and ion identification. We conclude with a discussion of the results.

## 2. Matrix assisted laser desorption and ionisation-time of flight mass spectrometry (MALDI-TOF MS)

In recent years, MALDI-TOF has gained greater attention from proteomic scientists as it produces high resolution data for proteome studies. There are three main challenges for mining the MALDI-TOF MS data. Firstly, the data quality of MALDI-TOF is very much dependent on the settings of the instrument. These settings include user-controlled parameters, i.e. deflection mass to remove suppressive ions and the types of calibration used for peak identification; and instrument-embedded settings, i.e. the time delayed extraction which is automatically optimised by the instrument from time-to-time based on the preset criteria in the instrument, peak identification protocols in the calibration and the software version used to generate and to visualise MS data. These settings have been altered, by either different users or by the instrument, to optimise detection of as many peptides as possible for each experiment. Table 1 presents the implications of some of the different instrument settings that may affect the quality of the final MS spectra.

**Table 1:** Examples of the experiments conducted using control samples with different settings applied in the MS instrument

Sample group	Total samples	Deflection mass (user-controlled)	Delay time (instrument-controlled)	Calibration standard (user/instrument-controlled)	Total *m/z *points	Intra-sample variation (in-between *m/z *ranges 800-3500)
Control (Plate 1)	15	650 da	9993 ns	Internal	198592	95223 points ± 824
Control (Plate 2)	21	650 da	9993 ns	Internal	198592	95213 points ± 3
Control (Plate 3)	10	450 da	9999 ns	Internal	198584	95200 points ± 825
Control (Plate 4)	16	450 da	9999 ns	External	198584	95199 points ± 3
Control (Plate 5)	10	450 da	10003 ns	External	198602	95211 points ± 3

When different settings were used to process biological samples, the mass assignment of a given *m/z *point will be shifted, in effect, causing a shift in mass accuracy through a population. Although these variations are mainly caused by other mechanical settings, such as the spotting pattern, instrument temperature, laser power attenuation and calibration constants; the lack of a standard protocol on the user-controlled setting will further contribute to noise in the data. This makes the reproducibility of MALDI MS data low resulting in difficulties in the analysis of consistent signals through a population. In addition to these settings, parameters such as mass detection range, sample resolution (sample acquisition rate in GS/s) and the laser firing rate; as well as the way the sample being prepared, i.e. homogeneity of crystallisation of the sample on the target plate, may also affect quality of the finished MS data.

Secondly, the raw MALDI-TOF MS data contains high dimensionality data with a small sample size - a hallmark for genomic and proteomic data. Each raw spectrum contains tens to hundreds of thousands of *m/z *points, each with a corresponding signal intensity. Each *m/z *point in the raw spectral data merely represents a point in the signal wave which contains little or no biological insight. Prior to the availability of bioinformatics analysis, the candidate marker ion selection was performed based on visual inspection for each sample over a population, thus, leading to the high potential for human error and user bias, subsequently introducing flaws into the reported results. Such problems pose challenges to the use of machine learning methods for ion (peak) selection from raw MS data.

Thirdly, existing MALDI preprocessing techniques involve different mathematical approaches in different machine learning methods. Unlike in genomics, the ideal preprocessing techniques in proteomics is to effectively remove all types of uncertainty in the raw MS data so that data reproducibility and spectral comparison can be performed. A lack of standard procedures for "cleaning" the raw MS data results in several preprocessing steps and different techniques were applied in these steps. Some examples include the use of 5-step data preprocessing, i.e. smoothing, baseline correction, peak identification, normalisation and peak alignment, prior to peak selection and classification for MALDI-TOF MS data [8]; background noise filtering and data normalisation for SELDI-TOF MS data [3]; window-shifting binning and heuristic clustering to align ESI Micromass Q-TOF MS data [12]; wavelet transform filtering to separating background noise from the real signals for MALDI-TOF MS data [15] and SELFI-TOF MS data [16]. As a consequence, preprocessing MS data is complicated and the preprocessing step is vague.

Rather than further complicated the MS data analysis with complex steps in data preprocessing technique, we propose a simple and effective preprocessing method to preprocess high resolution MALDI-TOF-MS data. For our preprocessing technique, we measure peak regions of MALDI-TOF MS spectral using a standard average function applied to whole population of samples within the data.

## 3. Data sets

Two in-house raw MALDI-TOF MS data sets, each representing different sample types (i.e. serum and plasma), were used. These data sets comprised melanoma sera data categorised into stage 2 and stage 3 diseases, and cord blood plasma labelled based on the quantity of CD-34 positive stem cells (High versus Low).

All clinical samples analysed as part of this study were collected under the appropriate consent and given ethical approval.

### 3.1 Sample Preparation

The collected plasma and serum samples were stored at -80°C until analysis. The samples were diluted 1 in 20 with 0.1% Trifluoroacetic acid (TFA) before undergoing C_18 _clean up The reproducibility of Millipore C_18 _ZipTip refinement of blood derivatives has been previously reported [17,18]. C_18 _ZipTips (Millipore) were conditioned on a robotic liquid handling system (FluidX XPS-96 for the cord blood plasma samples or Proteome Systems Xcise for the melanoma serum) using 3 cycles (aspirate and dispense) of 10 μL 80% acetonitrile, followed by 3 cycles of 10 μL 0.1% TFA. Sample binding consisted of 15 binding cycles of 10 μL, followed by 3 wash cycles of 10 μL 0.1% TFA and 15 elution cycles of 8 μL of 80% acetonitrile. The eluted fraction was combined with ammonium bicarbonate (16.6 μL of 100 mM), water (7.6 μL), and trypsin (0.7 μL of 0.5 μg/μL, Promega Gold dissolved in ammonium bicarbonate) and incubated at 37°C overnight. The reaction was terminated with 0.5 μL of 1% TFA. Following this the samples underwent a second ZipTip clean up (as previously) and 1 μL of the eluate mixed with 1 μL of CHCA matrix and spotted directly onto a Bruker 384 spot ground steel MALDI target for analysis.

### 3.2. Melanoma data set

Melanoma serum samples were selected from a frozen collection of sera banked at Heidelberg University, Germany in the period from April 2002 to November 2004. The pre-banked samples were made available via a collaborative study with Heidelberg University. One hundred and one adult patients (58 males and 43 females) with histologically confirmed as melanoma stage 2 (S2) or stage 3 (S3) sera were analysed, yielding mass spectral data for 99 samples (49 samples in S2 and 50 in S3). Each sample contains 198597 *m/z *points.

### 3.3. Cord blood data set

Cord blood plasma was collected from Banc de Sang i Teixits (BTS), Barcelona and shipped to the Anthony Nolan Trust cord blood bank at Nottingham Trent University. We labelled the samples into two groups - Low (< 30 CD45 sidescatter low/CD34+ stem cells/μL blood) and High (~100 cells/μL) stem content. This collection of plasma produced 158 samples, each associated with *m/z *points varies from 114603-114616. Among 158 samples, 70 samples were categorised as containing a "High" number of stem cells and the remaining 88 samples with a "Low" number of stem cells.

## 4. Methods

### 4.1. Data preprocessing

**Figure 1:**
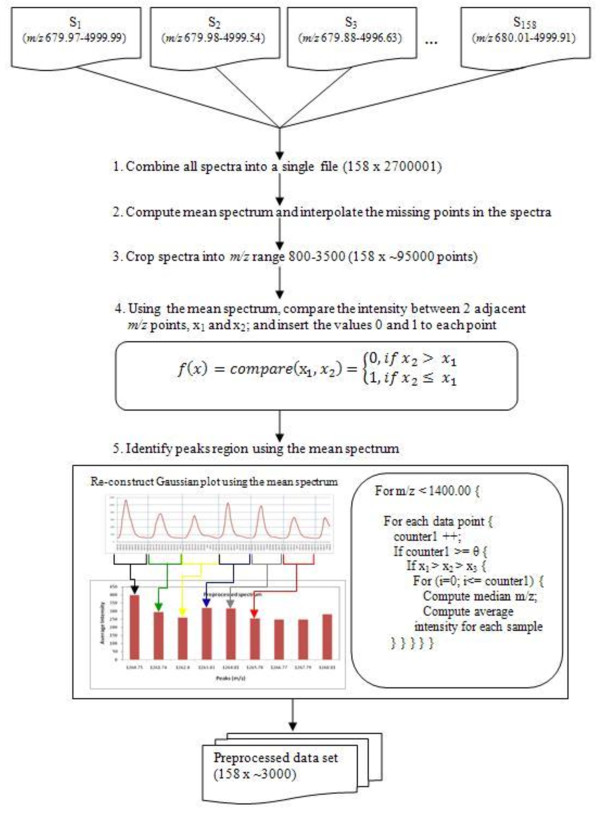
**Schematic illustration of data preprocessing step**.

The proposed data preprocessing technique is based on the Occam's razor principle to avoid any unnecessary complexity applied to the complex MS data. We used SpecAlign software [11] for data value imputation and average spectrum computation. Using the average spectrum, we re-construct the peak regions for all spectra in the population. Figure 1 outlines the workflow of our data preprocessing approach.

As illustrated in the figure, individual sample data were first merged into a single file according to the identical *m/z *points presented across the whole population. The interpolation function, based on a polynomial distribution function (SpecAlign software), was applied to insert missing values for missing *m/z *points in the spectra. An average spectrum was then computed and the *m/z *range 800-3500 is cropped for analysis in the next phase. This yielded a smaller data dimension approximately 95000 *m/z *points, from the original 2700001 points.

Using the average spectrum, we then compared the intensity of two *m/z *points and assigned the values '0' or '1' to indicate the increase or decrease respectively to the next adjacent *m/z *point in the merged file. Each time, 2 *m/z *points were used for comparison. This process continued until there were no more adjacent *m/z *points for comparison. The objective of such comparison was to reconstruct a Gaussian plot based on the spectral signal across a population of spectra and to further determine the region where a peak starts and ends. This point is worth emphasising as it simulates what is actually seen by the proteomic scientists and subsequently, avoid any form of confusion on the subject. This graph reconstruction could also minimise the risk of assigning a peak region to the wrong bin. We deliberately use very simple mathematical functions (i.e. mean and median) to avoid the possibility of a sophisticated mathematical formula complicating MS data preprocessing. From this reconstructed plot, we observed the pattern on both-tail (lower and upper boundary of a peak region) of the curve and defined the adequate criteria based on the observation. These criteria take account of the signal magnitude (peak size) and the maximum number of *m/z *points in the peak region (*m/z *value). Using these criteria, we identified the peak region, binned the *m/z *points within the region and standardised the peaks using the median *m/z *value in each region. The average intensity value of the region for each sample is used as the final values in the samples. This data preprocessing step has identified approximately 3000 peaks for both MS data sets.

#### Peak region identification

MS data is extremely complex and there is the possibility of a given peak potentially containing multiple peptide elements. There are also potential mass drift problems over multiple samples. Thus we defined peak regions based on the global average spectrum, computed from all of the samples in the population; rather than using the average spectrum computed from samples within the class. This global mean computation approach provides full information on the pattern of signal processing as it takes account of every intensity value appearing in the identical *m/z *points, regardless of the class that the sample belongs to. Consequently, the implication of sample size effects in statistical pattern recognition is significantly reduced and better accuracy on mass range assignment can be achieved. However, a significant drawback of using the global mean is that the accuracy of the pattern recognition in the signal processing will be severely affected by outliers and this leads back to the question on the quality of the MS data being analysed.

To alleviate the mass drift problem, we computed the global average spectrum using interpolation function in SpecAlign software. This interpolation function has embedded smoothing technique which automatically pre-filtered the data with 0.2 Da bin size. Using the average spectrum, we then constructed a Gaussian plot represent signal patterns in the population.

We observed a similar signal wave pattern on the average spectrum for both the data sets. A long, uninterrupted sequence of '0' value were found in each peak region in the average spectrum provides us the cut-off proximity for lower boundary between peak regions. When we visualised data values into a Gaussian plot, we observed that a peak would normally begin with at least 3 consecutive '0' values (the left-tailed of a curve). Thus, we defined the lower boundary of a peak region based on the presence of at least 3 consecutive '0' values.

To define the upper boundary of a peak region, we take into consideration of signal distortion and condition of the instrument. Observations on the upper boundary in the Gaussian graph (the right-tailed of a curve) of the signal pattern for every 1000 Da were performed. We observed that the variability on the signal (i.e. broader wavelength) and the presence of mechanical noise on 5 *m/z *checkpoints, i.e. 800.00, 1400.00, 1900.00, 2400.00 and 3000.00. Using these checkpoints, we defined the upper boundary of a peak region based on the minimum number of sign '1' (i.e. decrement signs) to be presented in each checkpoint.

### 4.2 Candidate marker ion identification

**Figure 2:**
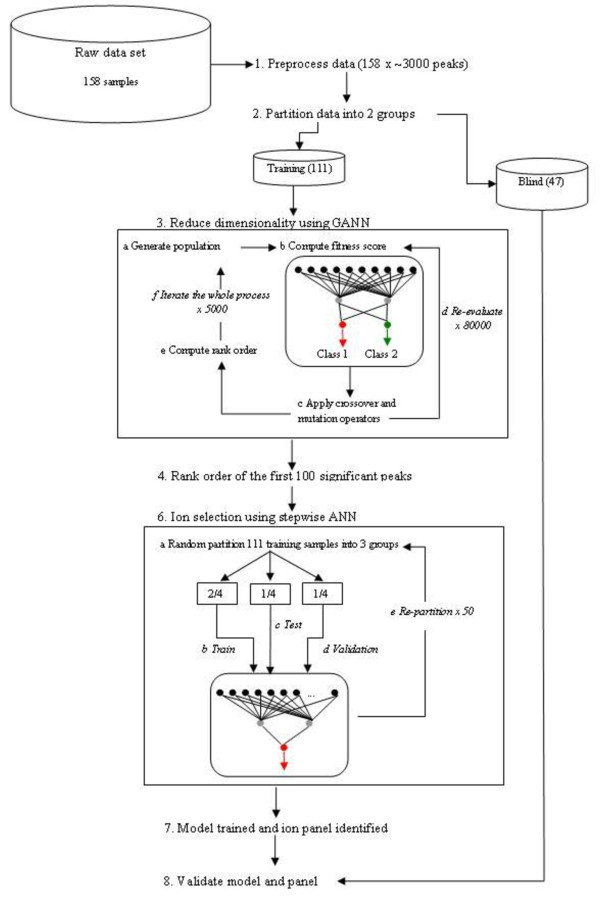
**Schematic illustration of ion identification analysis for MALDI-TOF MS protein profiling**.

As illustrated in Figure 2, we first preprocess the raw MS data. The data preprocessing steps was elaborated in length in the previous section. The data was then split into training and blind sets based on a ratio of 70:30, i.e. 70% for model training and the remaining 30% as a complete blind set to evaluate the performance of the model. A hybrid genetic algorithm-neural network (GANN) algorithm was used to filter the training set to identify a more focused subset of significant peaks. This peak subset was then analysed using the stepwise artificial neural network (ANN) to identify the most important peaks based on their predictive performance. This was represented by a rank order. In the stepwise ANN, the training set was further split into 3 groups, with the ratio of 60:20:20. A 60% of the data is used for training the network, 20% for testing (i.e. early stopping criteria based on mean squared error (MSE) for ANN) and the remaining 20% for validating the model. We re-sampled the data 50 times randomly to obtain an unbiased panel of significant ions. Finally, we validate our panel using the blind set. Subsequent sections discuss GANN and stepwise ANN.

#### 4.2.1. Data reduction using genetic-algorithm-neural network (GANN)

Genetic algorithm-neural network (GANN) is the bespoke hybrid genetic algorithm (GA) and artificial neural network (ANN) program that was developed for microarray analysis [19-21]. The GANN algorithm is a form of co-evolution of two distinct objectives, i.e. to find feature subset that enable an accurate classification for high dimension data. To do so, GANN utilised the universal computational power of ANN to compute the fitness score for GA and at the same time, GA optimises the ANN weights. Further information on GANN algorithm can be found in our previous study [22]. Table 2 summarises the GANN parameters used in this paper.

**Table 2:** Summary of the GANN parameters

Parameter	Setting
Population size	300
Chromosome size	20 features
Chromosome Encoding	Real-number representation
	
Fitness Function	The total number of correctly labelled samples
Selection	Tournament, tournament size = 2
ANN architecture	20-2-2
ANN size	48 nodes including 4 bias nodes
ANN learning algorithm	Feedforward
ANN activation function	Tanh
	
Crossover operator	Single-point, P_c _= 0:5
Mutation operator	P_m _= 0:1
Elitism strategy	Retain N-1 chromosomes in the population, where N is the total number of chromosomes in the population
	
Evaluation size	80000
Whole cycle repeat	5000

#### 4.2.2. Ion identification and prediction using stepwise artificial neural network (ANN)

Stepwise artificial neural network (ANN) is another bespoke program that was developed for mass spectra analysis [23-25]. In the stepwise ANN model, a 3-layered network architecture with a backpropagation learning algorithm was developed to train the data sets. First, each variable (i.e. peak) from the data set was used as an individual input to the network to create *n *individual network models with the structure of 1-2-1. These *n *models were then trained using Monte-Carlo cross-validation process and random sub-sampling to create 50 sub-models for each *n *model. The objective of using such cross-validation and random sub-sampling processes is to produce an unbiased set of predictive error rate for each variable in the data set. These models were then ranked based upon their average predictive error rate from the test data from each sub-model. The model with the lowest average predictive error identified the most important single ion which was selected for inclusion in the subsequent additive step. Because of the incorporation of stepwise approach in our ANN algorithm, the whole modelling process was looped with an increment of 1 as the input nodes to the network architecture, i.e. 2-2-1 and so on. For each loop, the remaining inputs were sequentially added to the previous best input, creating *n*+1 models each containing two inputs, until the predefined number of steps is met. Further information on stepwise ANN algorithm can be found in our previous study [25]. Table 3 summarises the stepwise ANN parameters used in this paper.

**Table 3:** Summary of the stepwise ANN parameters

Parameter	Setting
ANN architecture	I-2-1. For each run, the increment of 1 node in the input layer, I
Search method	Stepwise
ANN learning algorithm	Backpropagation
ANN activation function	Tanh
Learning rate	0.1
Momentum rate	0.5
Maximum epochs	3000
Window epoch	1000
Threshold for error	0.01
Random sampling	50
Maximum repeats on stepwise	10
Maximum loops on the whole modelling process	10
Cross-validation	Monte-Carlo with the ratio of 60:20:20

## 5. Results

To evaluate the performance of our methods for preprocessing raw MS data and identifying candidate marker ions, the data was split into 2 groups, i.e. training and blind sets. The Monte-Carlo cross-validation (MCCV) was applied on the training set (as illustrated in Figure 2) and the validation was performed using a separate blind data set which is completely unknown to GANN and stepwise ANN. Table 4 summarises the data sets and the classification results based on the independent blind data sets.

**Table 4:** Summary of the data sets and the classification results based on 50 random sampling

Data set	Class	Sample type	Sample size	Total peaks	Training set (MCCV)			Blind data set
						
					Train	Test	Validation	
Melanoma	S2 v. S3	Serum	99	2560	41	14	14	30
Classification (%)					93.21	97.38	90.62	90.93
								
Cord blood	High v. Low	Plasma	158	2647	67	22	22	47
Classification (%)					96.45	96.73	91.18	92.34

### 5.1. Melanoma inter-stage differentiation

For the melanoma data set, high classification performance was achieved with a panel of 10 ions identified by our model. Table 5 presents the rank order of the identified ions based on the MSE values returned by stepwise ANN for each training subset. The *m/z *value 1531.6 which is the first-ranked by our model shows a significant discriminative power between classes. This ion alone provided a median accuracy of 93% (result not shown) with an average test error rate of 0.097. With the identification of second highly ranked ion, i.e. *m/z *value 2916.61, the average test error rate was reduced to 0.054 and perfect median classification accuracy was achieved. Results show the decrease in the test error rate with the increase of ions added to the panel. This suggested that the synergistic nature of these highly ranked ions creates a strong statistical discriminative power in differentiating stage 2 and stage 3 melanoma cancers, which may also provide biological insight on the inter-stage tumour development in metastatic melanoma. Results also show the possibility of local maximum phenomenon on the fourth identified ion, i.e. *m/z *value 1196.57, in which slightly increased test error rate on the subsequent identified ions.

**Table 5:** List of the top-10 ranked ions for melanoma data set

Rank	Ion (*m/z*)	*m/z *(start)	*m/z *(end)	Ave. Train Error	Ave. Test Error	Ave. Valid. Error
1	1531.6	1531.12	1532.08	0.099	0.097	0.110
2	2916.61	2916.12	2917.1	0.0656	0.054	0.074
3	2425.27	2424.79	2425.75	0.0605	0.049	0.070
4	1196.57	1196.1	1197.04	0.0485	0.041	0.065
5	2917.59	2917.14	2918.05	0.050	0.045	0.054
6	1940.05	1939.57	1940.51	0.047	0.048	0.060
7	2426.25	2425.78	2426.71	0.036	0.037	0.063
8	1995.99	1995.51	1996.47	0.047	0.044	0.065
9	2543.07	2542.58	2543.57	0.038	0.043	0.072
10	1197.56	1197.06	1198.05	0.050	0.049	0.076

**Figure 3:**
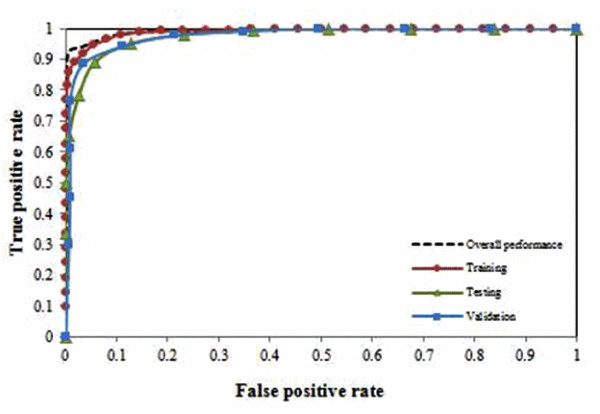
**ROC for model performance in the melanoma data set**.

We further examined the significance of these 10 ions using a set of 30 blinded samples on the previously trained 50 ANN sub-models (at 50 random sampling) and the rates for false positives (FPR) and true positives (TPR) were calculated. Table 6 shows the ANN prediction results based on the blind set. The blind set contains equal sample size for both classes, i.e. 15 samples for each class. Using the 50 trained ANN sub-models, we correctly classified 28 out of 30 blinded samples (90.93% classification accuracy). The TPR and FPR for S2 and S3 are 83.2% and 1.33%, and 98.67% and 16.80%, respectively. The receiver operating characteristics (ROC) based on the prediction performance of each training subset for 50 ANN sub-models was plot in Figure 3 and 3the area under ROC curve (AUC) is 0.991. The results show that the proposed preprocessing technique has successfully removing most of the noise from the original data and ions with high predictive power on classification have been identified from the preprocessed data.

**Table 6:** ANN prediction based on 30 blinded samples in the melanoma data set

	50 ANN sub-models					
					
Sample label	S2	S3	ANN output	Std err in 95% CI	ANN classification	Target output
Blind 1	50	0	0	0	S2	S2
Blind 2	50	0	0	0	S2	S2
Blind 3	50	0	0	0	S2	S2
Blind 4	50	0	0	0	S2	S2
Blind 5	0	50	1	0	S3*	S2
Blind 6	40	10	0.200	0.115	S2	S2
Blind 7	50	0	0	0	S2	S2
Blind 8	50	0	0	0	S2	S2
Blind 9	46	4	0.080	0.078	S2	S2
Blind 10	50	0	0	0	S2	S2
Blind 11	42	8	0.160	0.106	S2	S2
Blind 12	47	3	0.060	0.069	S2	S2
Blind 13	50	0	0	0	S2	S2
Blind 14	49	1	0.020	0.040	S2	S2
Blind 15	0	50	1	0	S3*	S2
Blind 16	0	50	1	0	S3	S3
Blind 17	0	50	1	0	S3	S3
Blind 18	0	50	1	0	S3	S3
Blind 19	0	50	1	0	S3	S3
Blind 20	0	50	1	0	S3	S3
Blind 21	5	45	0.900	0.087	S3	S3
Blind 22	0	50	1	0	S3	S3
Blind 23	0	50	1	0	S3	S3
Blind 24	5	45	0.900	0.087	S3	S3
Blind 25	0	50	1	0	S3	S3
Blind 26	0	50	1	0	S3	S3
Blind 27	0	50	1	0	S3	S3
Blind 28	0	50	1	0	S3	S3
Blind 29	0	50	1	0	S3	S3
Blind 30	0	50	1	0	S3	S3

S2 refers to the stage 2 of melanoma. S3 is the stage 3 of melanoma. ANN output is computed based on the average performance from 50 random samplings. Std err with 95% CI refers to the standard error for ANN output in 95% confident interval range. ANN classification indicates the final outcome of the model. Target output refers to the original group to which the sample belongs to.

### 5.2. Cord blood characterisation based on the quantity of stem cells

For the cord blood data set, our model has, again, achieved high classification performance with 10 significant ions identified from a pool of 2647 peaks. Table 7 presents the rank order of the selected ions for cord blood samples. Our model shows that the *m/z *value 2914.5 has discriminated, on average, 95% of the samples in the test set, with the average test error rate of 0.093. With the insertion of ions, i.e. *m/z *values 1062.6, 3058.5 and 1424.9, the average test error rate was significantly decreased to 0.029. This indicates that these ions are strong predictors for this data set. The test error rate is further reduced to 0.022 when all 10 ions were used on classification of 111 training samples.

**Table 7:** List of the top-10 ranked ions for the cord blood data set

Rank	Ion (*m/z*)	*m/z *(start)	*m/z *(end)	Ave. Train Error	Ave. Test Error	Ave. Valid. Error
1	2914.5	2913.96	2915.01	0.090	0.093	0.095
2	1062.6	1062.01	1063.28	0.033	0.030	0.042
3	3058.5	3058.04	3058.94	0.042	0.032	0.038
4	1424.9	1424.42	1425.42	0.030	0.029	0.047
5	3460.8	3460.32	3461.31	0.026	0.027	0.035
6	3061.5	3060.99	3061.92	0.027	0.025	0.045
7	2081.1	2080.59	2081.63	0.027	0.031	0.047
8	2369.3	2368.86	2369.75	0.023	0.025	0.050
9	1073.6	1073.19	1074	0.023	0.025	0.050
10	3062.4	3061.96	3062.93	0.019	0.022	0.032

**Figure 4:**
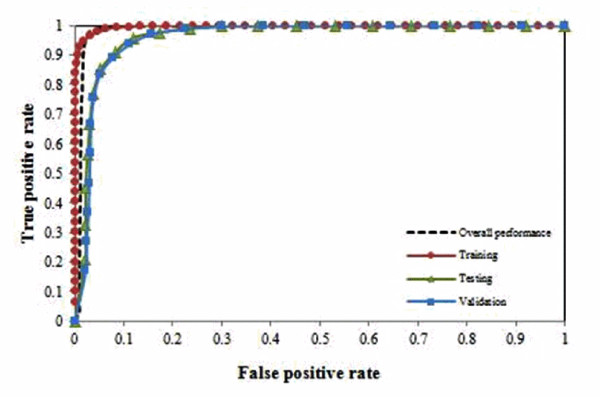
**ROC for model performance in the cord blood data set**.

We next computed the TPR and FPR of the model based on these 10 ions on a set of 47 blinded samples using previously trained 50 ANN sub-models. Table 8 shows the ANN prediction results based on the blind set. Among the 47 blinded samples, 20 samples in High group and the remaining 27 in Low group. Using the 50 ANN sub-models, we achieved classification accuracy of 92.34% on the blind set with only one misclassification. The TPR and FPR for the Low group are 92% and 7.23%; and 92.76% and 8% for the High group, respectively. We also plotted ROC, as showed in Figure 4. The AUC of the ROC curve is 0.986. This further supports our methods are robust for raw MS data preprocessing and significant ion selection.

**Table 8:** ANN prediction based on 47 blinded samples in the cord blood data set

	50 ANN sub-models					
					
Sample label	High	Low	ANN output	Std err in 95% CI	ANN classification	Target output
Blind 1	48	2	0.040	0.057	High	High
Blind 2	50	0	0	0	High	High
Blind 3	50	0	0	0	High	High
Blind 4	50	0	0	0	High	High
Blind 5	50	0	0	0	High	High
Blind 6	50	0	0	0	High	High
Blind 7	35	15	0.300	0.132	High	High
Blind 8	50	0	0	0	High	High
Blind 9	48	2	0.040	0.057	High	High
Blind 10	41	9	0.180	0.111	High	High
Blind 11	48	2	0.040	0.057	High	High
Blind 12	50	0	0	0	High	High
Blind 13	40	10	0.200	0.115	High	High
Blind 14	50	0	0	0	High	High
Blind 15	46	4	0.080	0.078	High	High
Blind 16	49	1	0.020	0.040	High	High
Blind 17	50	0	0	0	High	High
Blind 18	50	0	0	0	High	High
Blind 19	41	9	0.180	0.111	High	High
Blind 20	28	22	0.440	0.143	High	High
Blind 21	50	0	0	0	High	High
Blind 22	11	39	0.780	0.120	Low	Low
Blind 23	0	50	1	0	Low	Low
Blind 24	0	50	1	0	Low	Low
Blind 25	2	48	0.960	0.057	Low	Low
Blind 26	2	48	0.960	0.057	Low	Low
Blind 27	0	50	1	0	Low	Low
Blind 28	0	50	1	0	Low	Low
Blind 29	0	50	1	0	Low	Low
Blind 30	0	50	1	0	Low	Low
Blind 31	0	50	1	0	Low	Low
Blind 32	0	50	1	0	Low	Low
Blind 33	0	50	1	0	Low	Low
Blind 34	0	50	1	0	Low	Low
Blind 35	2	48	0.960	0.057	Low	Low
Blind 36	34	16	0.320	0.135	High*	Low
Blind 37	19	31	0.620	0.140	Low	Low
Blind 38	0	50	1	0	Low	Low
Blind 39	5	45	0.900	0.087	Low	Low
Blind 40	16	34	0.680	0.135	Low	Low
Blind 41	0	50	1	0	Low	Low
Blind 42	8	42	0.840	0.106	Low	Low
Blind 43	5	45	0.900	0.087	Low	Low
Blind 44	0	50	1	0	Low	Low
Blind 45	0	50	1	0	Low	Low
Blind 46	0	50	1	0	Low	Low
Blind 47	0	50	1	0	Low	Low

High and low refer to the quantity of stem cells in cord blood. ANN output is computed based on the average performance from 50 random samplings. Std err with 95% CI refers to the standard error for ANN output in 95% confident interval range. ANN classification indicates the final outcome of the model. Target output refers to the original group to which the sample belongs to.

## 6. Discussion

Unlike genomic data, raw MALDI-TOF MS spectral are characterised by a high dimension of noise caused by varying factors, from instrument settings, sample preparation, chemical noise, instrument temperature, and many more. As a result, a data preprocessing technique is usually required to convert the raw data into knowledge for further analysis. Currently, data preprocessing approaches for MS involve sophisticated mathematical understanding and multiple preprocessing steps. There is a lack of standard guidelines for performing these steps, and variation is introduced depending on user experience. Furthermore, existing publicly available MS preprocessing tools are designed for either SELDI MS or LC-MS use, rather than for MALDI-TOF MS use. Consequently, very limited functions of these tools can be used in MALDI-TOF MS data analysis. Thus, we have developed an in-house data preprocessing approach for removing inter- and intra-sample variability problems in raw MALDI-TOF MS data.

Our data preprocessing approach followed the Occam's razor principle, in which we deliberately used standard mathematical operators, i.e. mean and median, to compute average spectrum for all samples in the data sets. We utilised the interpolation function provided in SpecAlign software to alleviate mass drift problem. Based on this average spectrum, we re-constructed the signal pattern of the spectra and this re-construction provided the information on the peak regions that are likely to be appeared in all spectra across the population. We then applied a GANN algorithm to perform data reduction and a stepwise ANN algorithm for ion identification.

A potential difficulty with our data preprocessing approach is the choice of an appropriate mathematical operator to be used for intensity value computation for each peak region. We conducted 2 sets of experiments using 2 standard mathematical operators, i.e. mean and maximum operators. Using the maximum operator, we were not able to identify a strong predictive feature subset. We believe that this is due to data homogeneity caused by preprocessing (i.e. when the two classes are very similar having very few or no identifiable discriminating features), as we used only the maximum intensity values for each sample within a peak region and this lead to the equalisation of data in both classes. As a result, what was originally a strongest prediction feature became of equal significance to secondary or less significant features. To avoid data homogeneity, we have decided to use average function in this study. The average function takes account of every *m/z *point inside the peak region and this preserves predictive feature set within the data; however, a potential drawback is that noise still exists in the data. Therefore, we used GANN and stepwise ANN for feature selection and classification. GA and ANN are two widely used methods for handling noisy and complex data. We applied MCCV and random sampling techniques to minimise the risk of over-fitting in the ANN and to obtain unbiased rank order of the markers.

Another potential issue with our methods is elucidating its potency for identifying interesting features from the MS data. To overcome this problem, we produced a list of ions ranked by their significance (i.e. mean squared error) to the classification. Using this list, we observed that little or no improvement on the error after the first 10 ions. These ions have provided the potential for cost effective biomarker identification. Although for the melanoma data, the error started to increase after the first 7 ions, the increment is not obvious and it is difficult to draw a solid conclusion on whether or not it is a premature convergence (i.e. local maxima) or the model was over-fitted. When we validated these 10 ions using the complete blind data sets, we were able to correctly classify more than 90% of the blinded samples for both the data sets with reasonably low FPR and high TPR. For the melanoma data set, we obtained FPR of 1.33% for S2 and 16.8% for S3, based on the 10 ions selected by our model. For the cord blood data set, we achieved FPR of 7.23% and 8% for L (low) and H (high) groups, respectively. We also performed ROC analysis based on the classification performance of each sample in 50 random sampling and > 0.9 AUC values were achieved. This supports the potential use of our methods as a pre-screen to routine biomarker identification.

As the main purpose of this paper was to evaluate our methods for selecting statistically significant ions from high resolution MALDI-TOF MS data, we did not include biological assessment on the identified panels due to time and financial constraints.

We believe we have offered an alternative solution for the identification of candidate markers based on differential analysis of MALDI-TOF MS data. Our data preprocessing approach was simple and yet effective for removing most of the uncertainty values from the raw data. Our bespoke algorithms are robust for handling noisy data and cost effective for candidate marker selection. For future work, studies of biomarker validation on the identified panels will be performed to support our methods as a pre-screening method to routine biomarker identification.

## Competing interests

The authors declare that they have no competing interests.

## Authors' contributions

DLT: Developed analysis methodology; Analysed data; Wrote the manuscript. DJB: Prepared the samples; Performed the experiment; Analysed the data; Wrote the manuscript. CC: Prepared the samples; Performed the experiment; Analysed the data; Approved the final manuscript. JS: Prepared the samples; Performed the experiment; Approved the final manuscript. SGG: Contributed samples; Approved final manuscript. SQ: Contributed samples; Approved final manuscript. RR: Funded the experiments; Approved final manuscript. GRB: Developed analysis methodology; Approved final manuscript. All authors read and approved the final manuscript.
